# Typhoid Fever at Peterhead

**Published:** 1907-09-14

**Authors:** 


### Public Health and Hygiene.

### TYPHOID FEVER AT PETERHEAD.

A mild epidemic of typhoid fever has visited the northern town of Peterhead. According to the latest report of the Medical Officer of Health for the burgh, there have been notified altogether 195 cases. Dr. Dittmar, Medical Inspector of the Scottish Local Government Board, in an interim report states that the cases are not associated with any particular milk supply, and milk can therefore be excluded as the cause of the spread of the disease. The sanitary condition of the town is not such as in itself would give rise to an outbreak of enteric fever, and he regards the burgh water supply as the cause of the epidemic. It was interesting in this connection to note that not a single case of the disease has arisen at His Majesty's convict prison, which has a water supply of its own.

An examination of the sources of the water supply leaves no doubt that several of these are liable to gross pollution. This is no news to the Peterhead Town Council. They have for long been aware that their water supply was not above suspicion. Indeed, it is only fair to them to state that for a long time they have been deliberating on the steps to be taken to obtain a supply of water of assured freedom from pollution and sufficient in quantity for the growing population of the town. The present supply is obtained from a variety of sources. It is stored in reservoirs, but is delivered unfiltered to the town. Considering that for so long the sources have been regarded with suspicion, it is remarkable that the Town Council have not sooner had resort to such secondary protection as is afforded by sand filtration. It seems, however, that local authorities will not incur the burdens of ensuring a pure water supply until they have been read the lesson of a direct experience of the dangers of continuing the use of a polluted supply. Town after town courts this experience and gets it, and still there remain Councils unconvinced of the urgency of spending the requisite sums in time to prevent the ultimately inevitable catastrophe. When this comes, not only the local authority presses forward with feverish haste and its accompanying panic extravagance, but the Local Government Board begins to exercise the pressure which is only misapplied in the time selected for bringing it to bear. While the herrings are off the Aberdeenshire coast the normal population of Peterhead, about 12,000 persons, is said to be nearly doubled. The fishing population, and those directly dependent upon it, move southward with the herrings, and should this literally floating population become affected there is grave danger that the disease may be carried to the different fishing towns along the east coasts of Scotland and England, and indeed even to the western islands, from which many of the fisher folk are drawn. There is no greater mistake than to suppose that typhoid fever is solely a water-borne disease. To ignore the direct spread of the disease by infected persons and formites is to overlook an imminent danger when typhoid fever prevails.

